# Functional Performance of Chitosan/Carbopol 974P NF Matrices in Captopril Tablets

**DOI:** 10.1155/2016/3240290

**Published:** 2016-10-25

**Authors:** Yuritze Alejandra Aguilar-López, Leopoldo Villafuerte-Robles

**Affiliations:** Departamento de Farmacia, Escuela Nacional de Ciencias Biológicas, Instituto Politécnico Nacional de México, Ciudad de México, Mexico

## Abstract

Chitosan and Carbopol have been used to form a complex through an electrostatic interaction between the protonated amine (NH3^+^) group of chitosan and the carboxylate (COO^−^) group of Carbopol. In situ polyelectrolyte complexes formations based on the physical mixture of chitosan and sodium alginate were found and could be used as an oral controlled release matrix. The aim of this work is the assessment of a possible interaction between the particles of chitosan and Carbopol 974P NF that could modify their technological performance in captopril tablets. The drug and excipients were evaluated as mixtures of powders and tablets. The mixtures with captopril contained Carbopol 974P NF, chitosan, or a 1 : 1 mixture thereof with polymer proportions of 10%, 20%, and 30%. The evaluated parameters were the powder flow rate, the powder compressibility index, and the compactibility and release behavior of the tablets. The observed technological behavior points out to a greater interaction between the particles of polymers with different charge than between particles of the individual polymers. This produces more coherent matrices restricting more efficiently the drug dissolution, more coherent tablets with higher compactibility, and less flowing powder mixtures. All this, however, requires additional investigation to confirm the current results.

## 1. Introduction

Characterization of the functional performance of pharmaceutical excipients can not only be considered as a requirement but also provide data that can be predictive in nature regarding the performance of final dosage forms. This data may provide insight into how a material will behave in a given process or dosage form. The characterization of the components of the formulations may be beneficial in developing a design space or control strategies [1].

Controlled release formulations are used to overcome the drawbacks of immediate release formulations. Matrix system is the most widely used method for development of controlled release formulations due to its easy of manufacture. Different natural and synthetic polymers are used for controlled release matrix systems which have the property to extend the release of the drug from the matrix system [2].

Chitosan is a partially deacetylated form of chitin and has received attention as a new excipient or functional material of potential in the pharmaceutical industry. Chitosan displays good excipient properties as well as chemical and physical stability [3].

Chitosan exhibits properties such as biocompatibility, biodegradability, and low immunogenicity. Its high positive charge density confers mucoadhesive properties. Mucoadhesivity is a desired property for the delivery of drugs to mucosal tissues. Chitosan also has a very low toxicity [4].

The release of drugs from chitosan particulate systems involves three different mechanisms: erosion, by diffusion and release from the surface of the particle. The release of drug mostly follows more than one type of mechanism. In the case of release from the surface, particulate and adsorbed drug dissolves rapidly and it leads to burst effect when it comes in contact with the release medium [5].

Carbopol polymers and Noveon AA1 polycarbophil have been included in a variety of different tablet forms such as swallowable (peroral), chewable, buccal, and sublingual tablets, providing controlled release properties, bioadhesion, and good binding characteristics [6].

Carbopol 974P NF polymer is highly crosslinked and produces highly viscous gels with short flow. Carbopol 974P NF is an oral pharmaceutical grade of carbomers. It readily hydrates, absorbs water, and swells. These properties make it a potential candidate for use in controlled release systems.

An increasing amount of Carbopol 974P NF in tablets formulations results in a reduced rate of drug release and a linearization of the drug release curve. The release kinetics from carbomer matrix tablets can display a zero-order drug release. However, added excipients can have a dramatic effect on in vitro drug release profiles, commonly, an increased drug release rate [7].

Carbopol is a polyacrylic acid polymer, which shows a sol to gel transition in aqueous solution as the pH raises above its pKa (5.5). Chitosan is an amine-polysaccharide that is also pH dependent. At pH exceeding 6.2 it forms a hydrated gel like precipitate. When these two polymers are combined, the gel strength could be significantly enhanced. The application of this principle to a timolol ophthalmic solution produced an in situ gelling drug solution. The Carbopol/chitosan mixture was found to be more efficient in retaining the drug as compared to Carbopol solution alone [8].

Chitosan and Carbopol have been used to form a complex through an electrostatic interaction between the protonated amine (NH3^+^) group of chitosan and the carboxylate (COO^−^) group of Carbopol. The formed complex showed a similar release pattern to that of HPMC when compacted as theophylline matrix tablets. The main release mechanism was diffusional. The complex showed as advantage a reduced pH dependency of Carbopol [9].

Polyelectrolyte complexes are the association complexes formed due to electrostatic interaction between oppositely charged polycations and polyanions, avoiding the use of chemical crosslinking agents, thereby reducing the toxicity and the unwanted effects of the reagents [10].

In the same way, it was reported that chitosan-sodium alginate polyelectrolyte complexes could be used as an oral controlled release matrix. In previous reports, in situ polyelectrolyte complexes formations based on the physical mixture of chitosan and sodium alginate were found, avoiding the process of preparing polyelectrolyte complexes. In this sense, physical mixtures of chitosan-sodium alginate were studied as extended release matrices. Using trimetazidine hydrochloride as a model drug, it was found that sodium alginate alone produced a drug release after 2 h of 52%; once sodium alginate was mixed with chitosan the drug release decreased significantly, to 43% after 2 h [11].

The aim of this work is the assessment of a possible interaction between the particles of chitosan and Carbopol that could modify the technological performance of these polymers in a controlled release matrix, using as a model drug captopril.

## 2. Materials and Methods

### 2.1. Materials

The materials used in this study were Carbopol 974P NF obtained from Lubrizol Mexico, chitosan mesh 100 obtained from Pronaquim, Mexico, and captopril obtained from Química Alkano, Mexico.

### 2.2. Mixtures to Be Evaluated

The drug and excipients were evaluated as mixtures. Corresponding amounts of captopril and Carbopol 974P NF, chitosan, and a 1 : 1 mixture thereof were weighed to obtain 60 g of mixtures of captopril with polymer proportions of 10%, 20%, and 30%. The powders were transferred into a small V-type powder mixer and mixed for 30 min.

### 2.3. Compressibility Index: Bulk and Tapped Density

The equipment used to assess the powders densities is of our own fabrication and similar to that used to determine the tap density of powders, described in the Handbook of Pharmaceutical Excipients [12, 13]. The tapper was adjusted at a rate of 50 taps per minute and the graduated cylinder was elevated up to a height of 15 mm. This device uses a 100 mL graduated cylinder joined to a glass funnel with an orifice of 8.2 mm. The bulk (*ρb*) and tapped density (*ρt*) of powders were determined using the above-mentioned tapping machine (*n* = 5). The 100 mL measuring cylinder was filled with 30 g of sample. The volumes were recorded at the beginning (bulk volume) and after 10 taps. The process continued until three successive volume measurements remained constant (tapped volume). The bulk density was calculated as the ratio of mass and bulk volume while the tapped density was calculated as the ratio of mass and tapped volume. The registered results are the average of five repetitions with the same sample. The powders were sieved through a mesh number 20 after each repetition. Carr's Index or compressibility index (CI-%) was calculated according to the following equation [14]:

(1) $$\begin{array}{c} \text{Compressibility Index}=\frac{\left(\rho t-\rho b\right)}{\rho t*100}. \end{array}$$

### 2.4. Powders Flow Rate

The material is weighed (approximately 30 g) and its flow rate assessed using the equipment described to assess the powders densities. The sample is gently poured into the funnel whose bottom opening was blocked. While unlocked, the tapper starts the movement. The powder flows through the orifice of the funnel, falling to the bottom of the cylinder. The time it takes to move the total powder poured through the funnel is registered. The flow rate is calculated by dividing the sample mass by the time. The assay is repeated 5 times, sieving the powder through a mesh number 20 after each measurement. The average of the repetitions is taken as the flow rate.

### 2.5. Compactibility

Tablets weighing 200 mg were compacted for 20 s in a hydraulic press, at a series of compaction pressures from 27 MPa to 163 MPa and using 8 mm circular flat-shaped punch and die. Tablet crushing strength was measured in triplicate, registering the results as an average. For this purpose, a tablet hardness tester Erweka TBH30 was used. The procedure involved placing each tablet diametrically between two flat surfaces and applying pressure until the tablet breaks down.

### 2.6. Dissolution Test

Tablets obtained as described in the subtitle compactibility and compacted at a compaction pressure of 109 MPa were used to determine the dissolution behavior. The dissolution profile was carried out in a Hanson Research SR6 under sink conditions, for a period of 6 hours using a paddle apparatus at 50 rpm. 900 mL of HCL 0.1N was used as the dissolution medium. For each composition, a dissolution test was performed with three repetitions at 37°C. Each tablet was placed in a coil of stainless steel wire, to prevent sticking or floating. At predetermined time intervals samples were removed, filtered, and evaluated with a spectrophotometer (Beckman DU 650, *λ* = 208 nm). After each sample was removed, the same amount of liquid was replaced, thus maintaining the volume in the vessel. The dissolution profile was expressed as the percentage of drug released, based on the total tablet content after dissolution in a magnetic stirring device.

## 3. Results and Discussion

### 3.1. Effect of Carbopol 974P NF and Chitosan on Flowability of Captopril Powder Blends

Powder flow is critical during tableting, as powders must flow easily and uniformly into the tablet dies to ensure tablet weight uniformity and tablets with consistent and reproducible properties.

Propiconazole formulations containing 20% Carbopol have shown a poor powder flowability, indicating the need of glidants to make them processable. Carbopol was considered dysfunctional in this respect [15]. Contrasting, it has been observed that the fluidity of combined powders of lactose or potato starch with chitosan was greater than that of powder mixtures containing microcrystalline cellulose [16].

**Figure 1:**
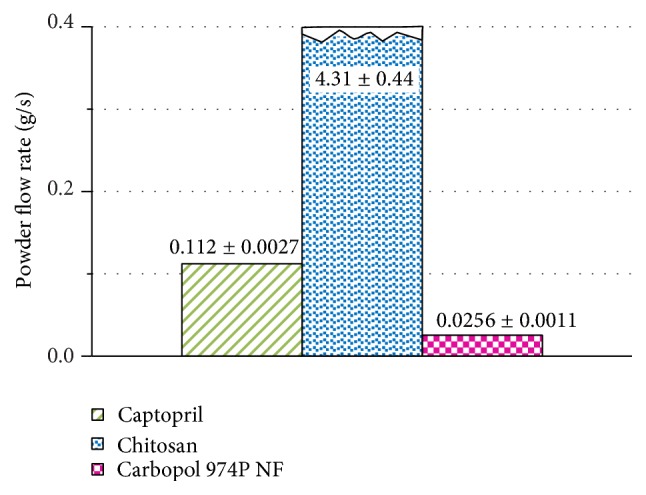
Powder flow rate through an orifice of 8.2 mm of captopril, Carbopol 974P NF, and chitosan.

Figure 1 shows the powder flow rate of captopril and the individual polymers, through a glass funnel with an opening of 8.2 mm and using a tapper as the driving force. The powder flowability of chitosan is quite superior when compared to captopril and Carbopol 974P NF. Chitosan is, according to Figure 1, a material hundred times more fluid than Carbopol 974P NF.

**Figure 2:**
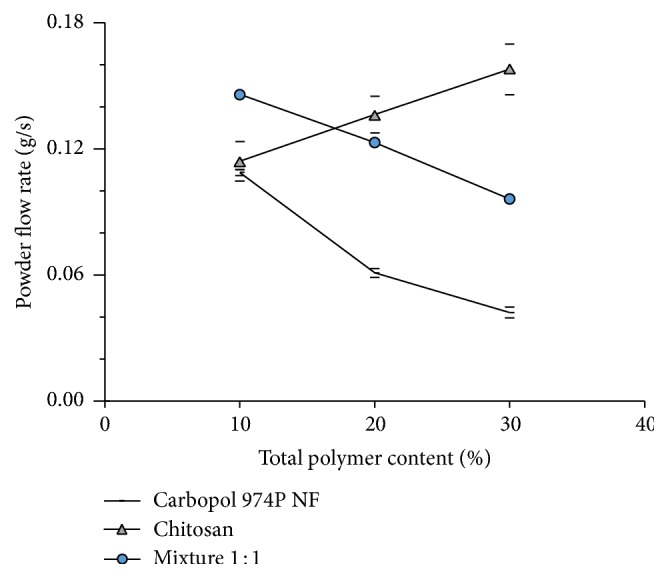
Effect of polymer proportion on the powder flow rate of admixtures with captopril, through a funnel with an opening of 8.2 mm (± St. Dev.).

Figure 2 depicts the effect of Carbopol 974P NF, chitosan, and the mixture thereof on the powder flow rate of captopril mixtures containing different polymer proportions. The results in Figure 2 confirm the in literature mentioned effect of chitosan. Chitosan increases the powder flowability of the mixtures as the proportion of this polymer increases.

Contrasting, increasing proportions of Carbopol 974P NF in the powder mixtures decrease their flowability. Powder blends containing the combination of chitosan and Carbopol 974P NF display powder flow rates with the same tendency to decrease the flowability as that shown by Carbopol 974P NF alone. However, the powder flow rates are higher. Chitosan improves the flowability of captopril blends with Carbopol 974P NF. So far, a special interaction between the polymers with different charge cannot be observed.

**Figure 3:**
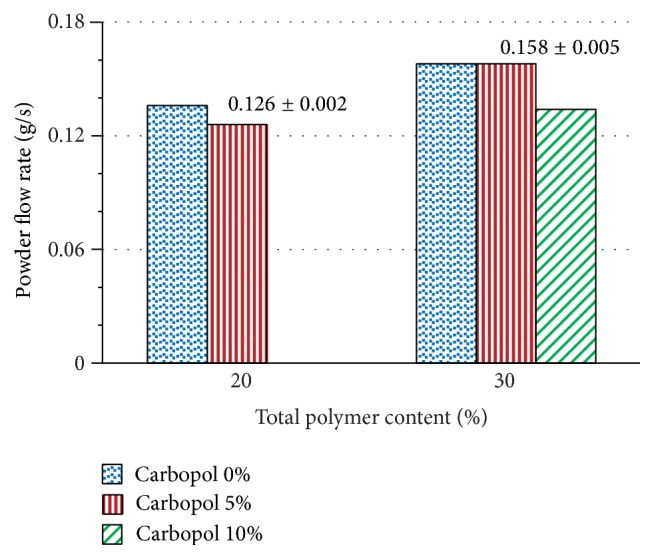
Effect of addition of Carbopol 974P NF on the powder flow rate of captopril/chitosan powder mixtures with a total polymer content of 20% and 30%.

Figure 3 shows the effect of adding Carbopol 974P NF on the powder flow rate of mixtures of captopril with chitosan. Powder mixtures with a total polymer content of 20% and 30% display a slight decrease in powder flow rate, when including 5% Carbopol 974P NF. Dilution of chitosan with captopril reduces drastically its powder flow rate. The subsequent addition of Carbopol 974P NF does not reduce further, in an important manner, the powder flow rate.

Bulk and tapped densities can provide information on flowability of powders when used to calculate the Carr Index. The lower the Carr Index is, the better the flowability of the powder is. According to the powder compressibility index, chitosans have been observed to display an average poor flowability (CI = 28) when compared to Avicel PH 200 (CI = 15) [17].

**Figure 4:**
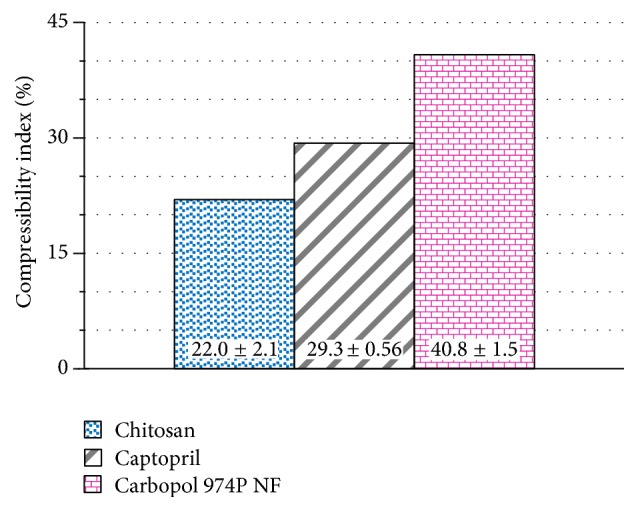
Compressibility index displayed by captopril, Carbopol 974P NF, and chitosan.

Figure 4 shows the powder compressibility index of captopril and individual polymers. As observed before by the powder flow rate (Figure 1), chitosan displays a lower compressibility index standing for a higher powder flowability. However, the difference in flowability between Carbopol 974P NF and chitosan is much smaller than that observed by the powder flow rate measurement (Figure 1). The powder flow rate of chitosan is more than one hundred times faster than that of Carbopol 974P NF. However, the compressibility index of Carbopol 974P NF is only twice that of chitosan. The compressibility index values stand for a chitosan powder flowability only two times greater than that of Carbopol 974P NF. The powder flow rate assessment expands the scale of powder flowability, making the differences between different powders more visible.

**Figure 5:**
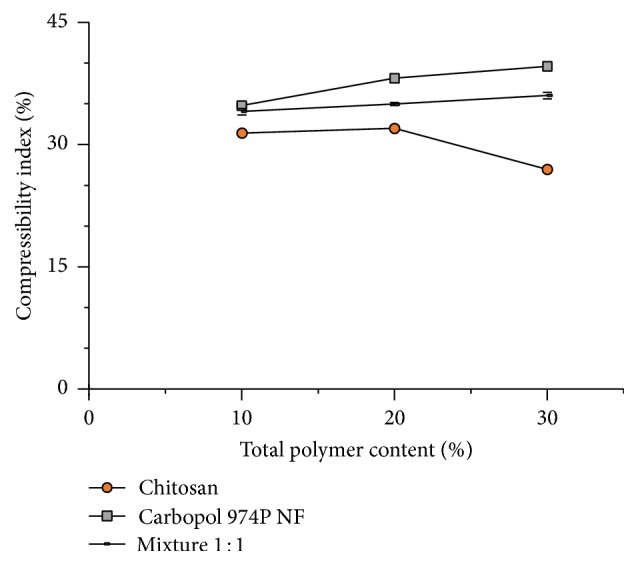
Effect of polymer proportion on the powder compressibility index of admixtures with captopril (± St. Dev.).

Figure 5 shows the compressibility index of captopril powder mixtures with different proportions of chitosan, Carbopol 974P NF, and the mixture thereof. Mixtures of captopril with chitosan show an average compressibility index of 30%, indicating a poor flowability. The original passable flowability of chitosan (Figure 4) decreases after mixing with captopril. However, mixtures with Carbopol 974P NF show an average compressibility index of 37.5%, which means a worse flowability than blends with chitosan. In addition, captopril mixtures with different proportions of the blend of equal parts of both polymers display an average compressibility index laying in between (35%).

The results observed in Figure 5 show the same trends observed earlier by the flow rate of the powders. The increase in proportion of Carbopol 974P NF in mixtures with captopril shows an increase in the compressibility index values, indicating a decrease of powder flowability. This occurs in the same way as observed before in a direct measurement of the powder flow rate (Figure 2). On the other hand, the increase in the proportion of chitosan in mixtures with captopril shows a decrease of compressibility index values, indicating an increase in flowability.

Captopril mixtures containing different proportions of the combination of the two polymers show a similar trend to that observed before by the measurement of the powder flow rate of mixtures containing only Carbopol 974P NF. The increase in the proportion of the polymer mixture increases the compressibility index values, indicating less fluidity. However, the compressibility index values of the mixtures of captopril with the combination of the two polymers are lower, indicating more fluid mixtures than those containing only Carbopol 974P NF.

The average value of the compressibility index of captopril mixtures containing individual polymers (33.8%) is less than the result observed with captopril mixtures with the combination of the two polymers (35.0%). This could indicate a higher adhesiveness between the particles of the polymer blend than between the particles of the individual polymers. However this would not be free of questioning given such a small difference.

**Figure 6:**
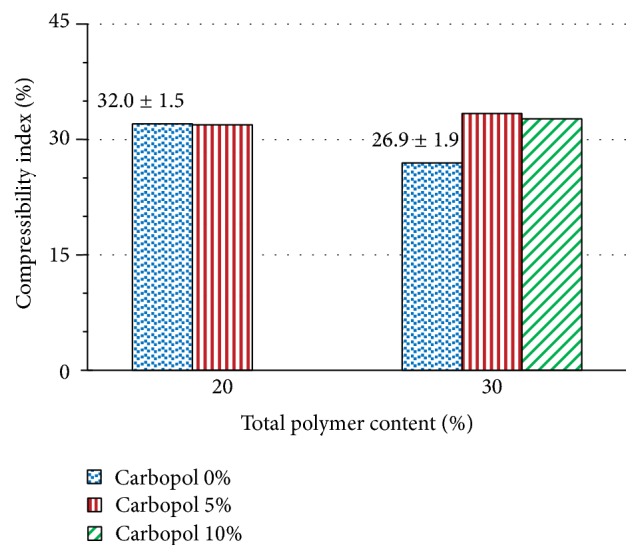
Effect of addition of Carbopol 974P NF on the powder compressibility index of captopril/chitosan mixtures with a total polymer content of 20% and 30%.

Figure 6 shows the effect of adding Carbopol 974P NF on the compressibility index of powder mixtures of captopril with chitosan. The powder mixtures with a total polymer content of 20% and 30% show a slight increase in the compressibility index of the powders when Carbopol 974P NF is included in the mixture. This indicates a slight decrease of fluidity, in the same manner previously observed in determining the flow rate of the powder (Figure 3).

### 3.2. Effect of Carbopol 974P NF and Chitosan on Compactibility of Captopril Tablets

Chitosan is known to reduce friction during tableting. The ejection force of lactose/chitosan tablets has been observed to be significantly smaller than that of lactose/microcrystalline cellulose [16].

Chitosans display deformation during compression combined with high elasticity after tableting. Chitosan shows tableting properties similar to those of microcrystalline cellulose (Avicel PH 200), although approximately 25% lower [17], even if the compactibility of chitosan has been reported to be less than a half of that of Avicel PH 102 [3]. The main difference with microcrystalline cellulose is that chitosan exhibits a high elastic recovery.

**Figure 7:**
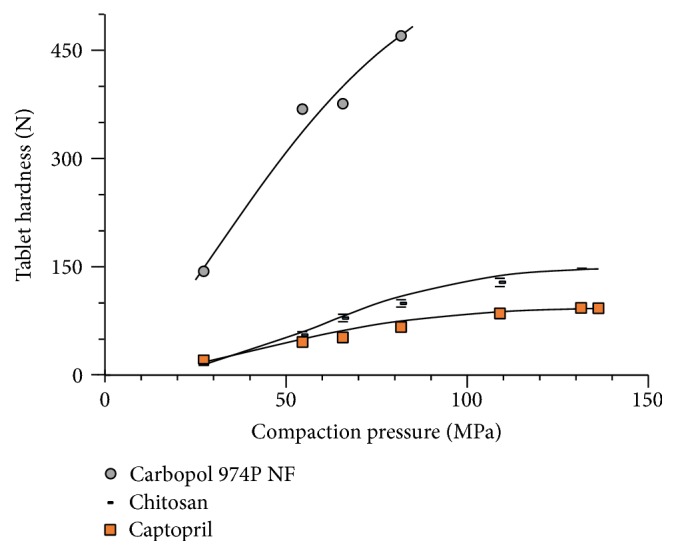
Compactibility curves of individual materials, captopril, Carbopol 974P NF, and chitosan (± St. Dev.).

Figure 7 depicts the compactibility of individual materials. Carbopol 974P NF shows a quite superior compactibility followed by chitosan and captopril. Considering the tablet hardness at a compaction pressure of 66 MPa, the compactibility of chitosan is 20% of that of Carbopol 974P NF while that of captopril is only 13%.

The points in Figure 7 are experimental and the lines are the calculated regressions with (2) [18–21].

(2) $$\begin{array}{c} \ln\left(\;-\ln\left(\;1-\frac{D}{D_{max}}\;\right)\;\right)=n*\ln\;Pc+I, \end{array}$$

where *D* is the hardness or crushing strength of the tablets, *D*_max_ is the maximum hardness attained by the tablets, *Pc* is the compaction pressure, *n* is the slope of the curve, and *I* is the intercept of the curve.

**Figure 8:**
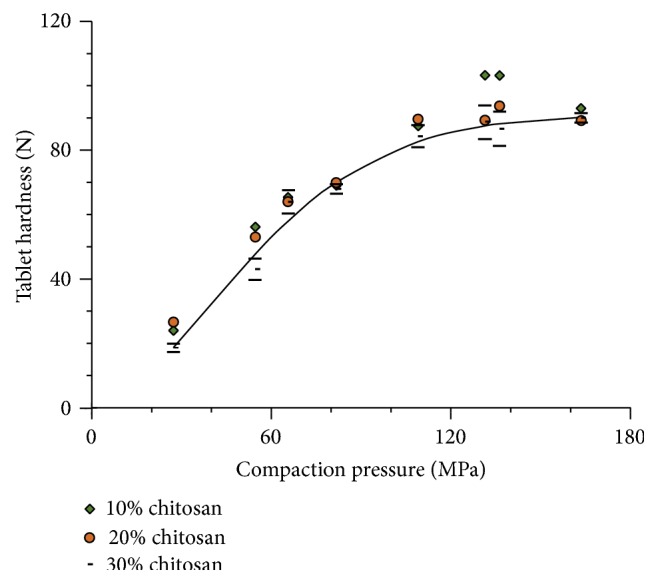
Effect of different proportions of chitosan on the compactibility profile of admixtures with captopril (± St. Dev.).

The compactibility of captopril admixed with different proportions of chitosan can be seen in Figure 8. The points are experimental and the curve for compatibility is that calculated for tablets containing a proportion of chitosan of 30%.

The experimental data depicted in Figure 8 do not allow the perception of different compactibility in captopril admixtures containing different proportions of chitosan. The influence of different proportions of chitosan on captopril compactibility is not appreciable. This can be ascribed to a small difference in compactibility between captopril and chitosan (Figure 7).

It has been observed that Carbopol forms tablets with higher hardness and lower friability than the known agglutinant PVP-K30 [6]. Carbopol produces metronidazole tablets with a crushing strength 3 times greater than tablets with the same proportion of hydroxypropyl methylcellulose [22].

**Figure 9:**
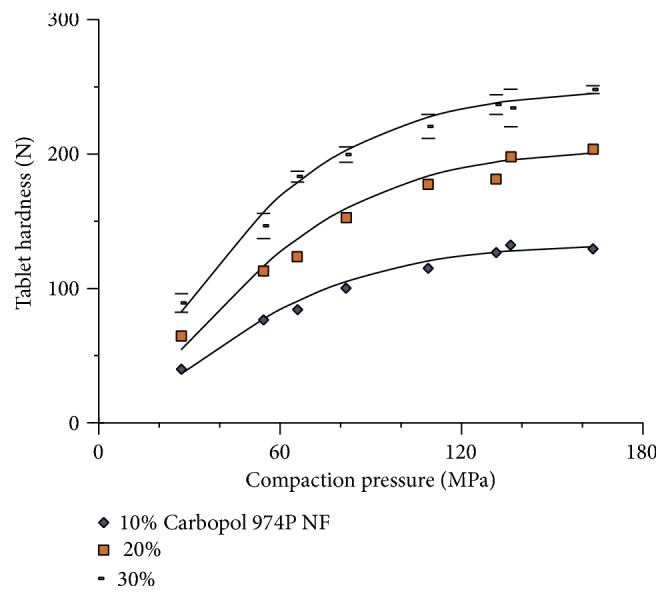
Effect of different proportions of Carbopol 974P NF on the compactibility profile of captopril tablets (± St. Dev.).

As can be seen in Figure 9, increasing proportions of Carbopol 974P NF produce tablets with an increasing tablet hardness. This significant progressive increase in compatibility is attributed to the high binding capacity previously shown by Carbopol (Figure 7). The increase in compactibility of captopril tablets occurs at the expense of losing compactibility of Carbopol.

**Figure 10:**
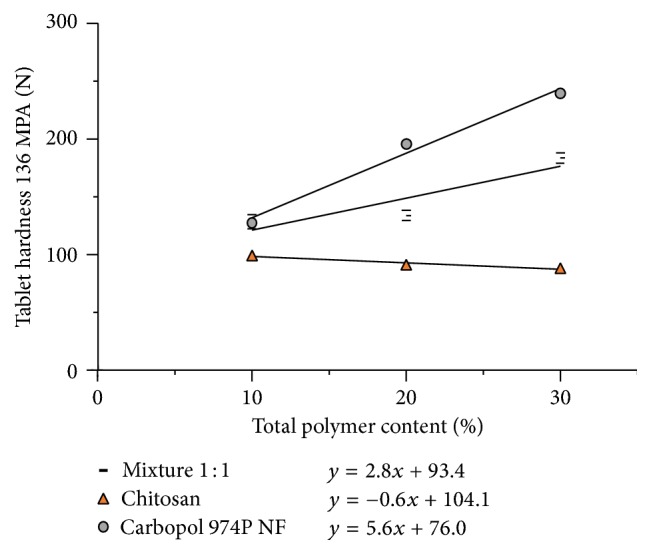
Effect of different polymer proportions on compactibility of captopril tablets calculated by regression at compaction pressure of 136 MPa (± St. Dev.).

The greater compactibility of Carbopol 974P NF over that of chitosan observed in Figure 7 is maintained after dilution of the two polymers with captopril, although the difference in compatibility is lesser. This can be seen in Figure 10. As can be seen in the figure, the effect of the polymer proportion on the tablet hardness of captopril tablets obtained at 136 MPa can be described with a linear relationship. Tablets containing chitosan exhibit a tablet hardness of 99 N at a polymer proportion of 10%. The tablet hardness decreases 0.6 N per unit percentage of chitosan added, as shown by the slope of the calculated regression line displayed in the figure.

On the other hand, captopril tablets containing Carbopol 974P NF begin with a higher tablet hardness (128 N) and display a slope indicating an increase in 5.6 N per unit percentage of the added excipient. The addition of Carbopol 974P NF and chitosan admixture increases captopril tablet hardness in the same way as does the Carbopol 974P NF, although less, 2.8 N per unit of percentage of added polymer mixture (Figure 10).

The hardness of tablets depicted in Figure 10 shows an average for captopril/Carbopol 974P NF admixtures of 189 N. This value can be taken as reference for the binding or agglutinant ability of the polymer. This binding ability is about twice that exhibited by chitosan admixtures (93 N). As could be expected, the use of a mixture of chitosan and Carbopol 974P NF produces tablets with a hardness in between (149 N). This last average value of the tablet hardness (149 N) is somewhat higher than the average hardness of the tablets obtained with the individual polymers (141 N). This result would be indicative of a special interaction between Carbopol 974P NF and chitosan that could be attributed to increased adhesiveness between these polymers because they have different charges.

**Figure 11:**
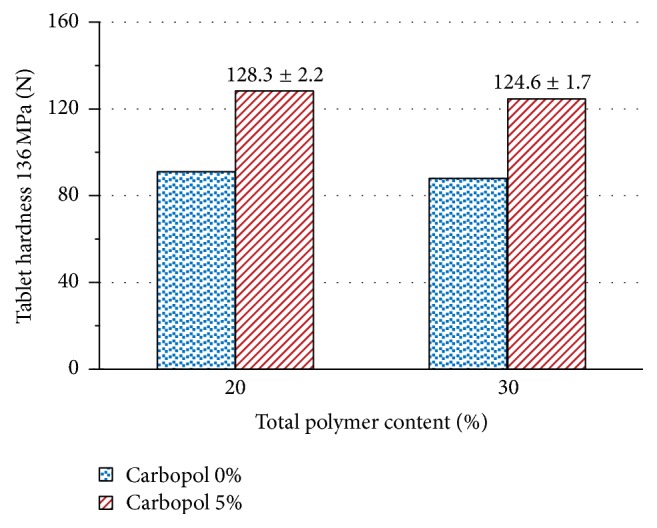
Effect of addition of 5% Carbopol 974P NF on the tablet hardness of captopril/chitosan tablets with a total polymer content of 20% and 30%.

Figure 11 shows the influence of addition of Carbopol 974P NF on compactibility of captopril/chitosan tablets, calculated by regression for a compaction pressure of 136 MPa. Tablets with a total polymer content of 20% and 30% display an increase in compactibility of approximately 40%, after inclusion of 5% Carbopol 974P NF.

### 3.3. Effect of Carbopol 974P NF and Chitosan on the Release Profile of Captopril Tablets

Drug dissolution from solid dosage forms is described with kinetic models in which the amount of drug dissolved is a function of time. In most cases, there is not necessarily a theoretical concept behind the mathematical models and empirical equations can be used to properly describe the behavior of drug release [23].

**Figure 12:**
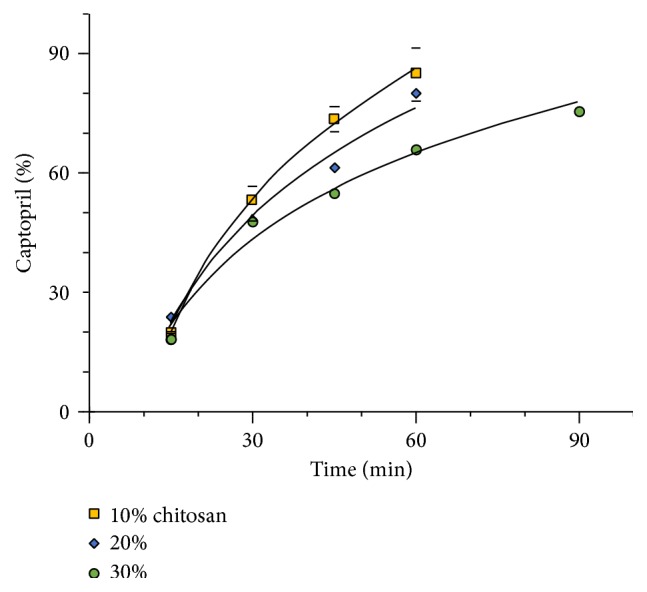
Effect of different proportions of chitosan in the dissolution profile of tablets of captopril (± St. Dev.).

Figure 12 depicts the release profile of captopril from chitosan matrices containing different polymer proportions. The figure represents the experimental points and the calculated regressions with a logarithmic model. The effect of chitosan is an increasing restriction of drug release as the polymer proportion in the tablets increases. The restriction of drug dissolution produced by chitosan, even if significant, cannot be considered sufficient for a controlled release formulation.

Carbopols produce in many cases a linear release kinetics; however, the release kinetics is also dependent of the polymer proportion and added excipients. It has been observed that different proportions of Carbopol 974P between 10% and 30% produce drug release profiles with different degrees of curvature. Moreover, the addition of coexcipients changed the release profile towards higher degrees of curvature, away from a desired zero-order or linear release [7].

**Figure 13:**
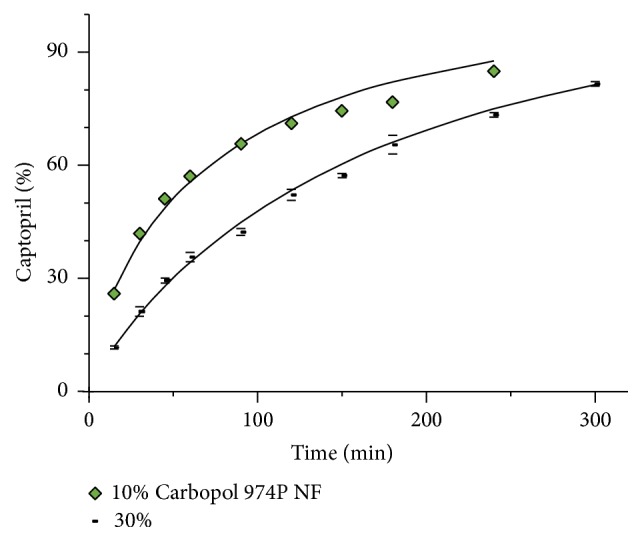
Effect of different proportions of Carbopol 974P NF on the dissolution profile of captopril tablets (± St. Dev.).

Figure 13 depicts the effect of different proportions of Carbopol 974P NF on the release profile of the tablets of captopril. As mentioned above, the degree of curvature of the release profile is dependent of the polymer proportion. Higher polymer proportions produce release profiles with a greater restriction of drug release and a lesser degree of curvature.

The release curves shown in Figure 13 are the result of a faster drug dissolution compared to hydration of the polymer and the subsequent binding of its particles. Once the gel layer has been established the release rate decreases [22]. The use of larger proportions of polymer increases the number of particles in the matrix, which facilitates their interaction and binding. This circumstance decreases the free drug dissolution.

Among the technological variables that influence the release from hydrophilic matrices, the use of polymer blends is a potential way of achieving the desired release properties [24]. Mixtures of different proportions of polymers with different permeation characteristics could provide a wide variety of release rates of a drug, due to changes in diffusivity of the drug through the polymeric barrier [25].

**Figure 14:**
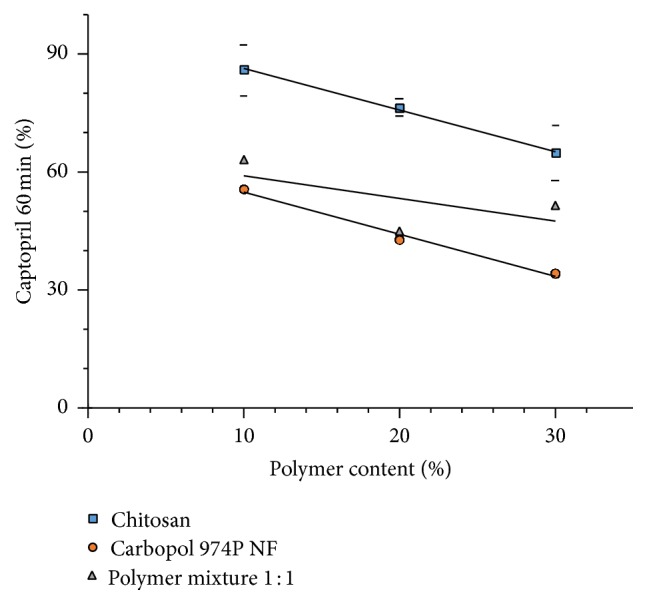
Effect of different proportions of Carbopol 974P NF, chitosan, and their 1 : 1 admixture on the calculated captopril dissolution at 60 min (± St. Dev.).

Figure 14 depicts the effect of different proportions of the polymers Carbopol 974P NF, chitosan, and a mixture thereof on the amount of captopril dissolved at 60 min. The admixture of Carbopol 974P NF with chitosan results in an intermediate release rate, when compared with release from matrices with individual polymers.

As shown in Figure 14, the use of the polymer blend does not allow a clear perception of additional interaction between polymers because they have different charges, even if the restriction of the dissolution of captopril, produced by the use of the polymer blend, is somewhat greater than the average release produced by individual polymers.

Taking the dissolution data of Figure 14 as a whole, matrices with chitosan display an average dissolution of 75.6% at 60 min. In the same way, the matrices with Carbopol 974P NF have an average dissolution of 44.2%. Taking these data as reference it might be expected that matrices containing equal parts of both polymers show an average dissolution of 59.9%. However, this is not the case. The average dissolution of the matrices containing the polymer mixture is 53.3%. This is 6.6% less dissolved captopril than might be expected. This means that the observed dissolution might include something more than the simple average of the two polymers with different permeation characteristics, possibly, a stronger interaction between polymer particles with a different charge. This interaction would produce matrices with greater coherence that restrict the dissolution of captopril more efficiently.

**Figure 15:**
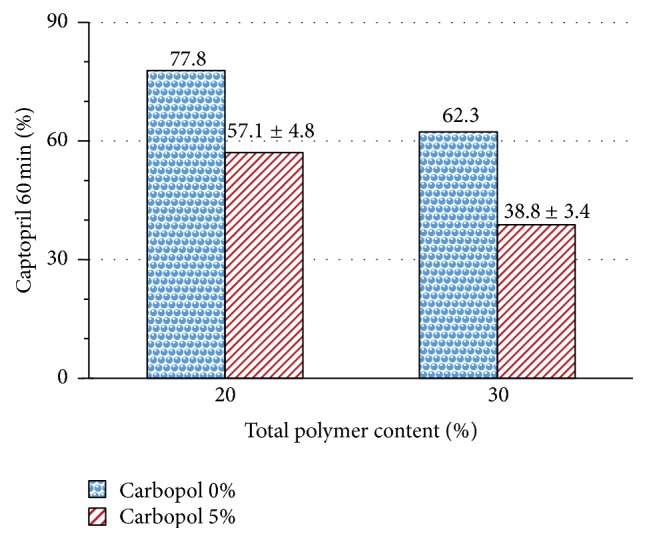
Effect of containing 5% Carbopol 974P NF on captopril dissolved in 60 min, from chitosan matrices with a total polymer content of 20% and 30%.

Figure 15 shows the influence of the addition of Carbopol 974P NF on captopril dissolved at 60 minutes, from matrices with chitosan. Matrices having a total polymer content of 20% and 30% show a reduction of captopril dissolved after 60 minutes of about 20%, when its content in Carbopol 974P NF is 5%.

## 4. Conclusion

Chitosan matrices exhibit about 40% higher captopril dissolution than Carbopol 974P NF matrices while the dissolution allowed by the polymer blend is approximately 6% lesser than the calculated average of dissolution of individual polymers. This can be possibly ascribed to a stronger interaction between particles with different charges producing more coherent matrices that restrict more efficiently the captopril dissolution.

The compactibility or agglutinant properties of Carbopol are twofold greater than those of chitosan. The compactibility obtained with the polymer mixture is something greater than the average compactibility of the tablets containing the individual polymers, pointing out to a greater adhesiveness between particles of polymers with different charge.

The compressibility index of captopril mixtures with Carbopol 974P NF is about 20% higher than that of mixtures containing chitosan. Captopril containing the polymer mixture displays a compressibility index higher than the average observed by powders containing the individual polymers. This indicates a possible greater adhesion between particles of different polymers than cohesion between the particles of single polymers.

The observed technological behavior of mixtures of Carbopol 974P NF with chitosan points out to a greater interaction between the particles of polymers with different charge than between particles of the individual polymers. This produces more coherent matrices restricting more efficiently the drug dissolution, more coherent tablets with higher compactibility, and less flowing powder mixtures. All this, however, require additional investigation to confirm the current results.
